# Diagnosis of Diseases of the Stomach

**Published:** 1900-03

**Authors:** Charles D. Aaron

**Affiliations:** Detroit, Mich., Consulting Gastro-Enterologist to Harper Hospital, Lecturer on Dietetics in the Detroit College of Medicine, etc., etc.


### DIAGNOSIS OF DISEASES OF THE STOMACH.*

By CHARLES D. AARON, M.D., DETROIT, MICH.,
Consulting Gastro-Enterologist to Harper Hospital, Lecturer on Dietetics in the Detroit College of Medicine, etc., etc.

The recognition of signs or symptoms by which one disease is distinguished from another is known as diagnosis. Every item must be taken into consideration before we are able to recognize the special form of disease. In the history of medicine, therapeutics began as a practical art, but with the progress of rational therapeutics and the displacement of empiricism, a need for accurate diagnosis has become more and more apparent. In fact, those who were treating disease empirically could not help realizing that the application of an adequate therapeutics depends upon an exact knowledge of the disease. The saving of unfortunate fellow men is our motive in administering to the sick, and no genial guest goes half so far as a scientific fact. In former days some persons were credited with special talent for curing the sick, and the practice of medicine was relegated to the priests as one of their functions. They observed and studied the sick from an objective standpoint, and they could not help but notice in a large number of patients certain resemblances as to the character and course of disease. They observed also that certain symptoms indicated a favorable and others unfavorable development. Some drugs prove efficacious in certain diseases, while others that proved to be without effect were excluded. In this way the first step was made toward the development of scientific diagnosis. A disease can be understood only when we have a correct conception of disease in general. This latter depends, of course, upon the study of pathology and upon the experience at the bedside, and cannot be acquired unless the physician is a careful observer and can differentiate between the essential and non-essential manifestations. This diagnostic tact, as I might call it, can be acquired through industrious study alone. The only correct way to assure ourselves of the form

*Read before the Northern Tri-State Medical Association at Adrian, Mich., January 16, 1900.

of the disease we come upon is to determine what the distinct phenomena in the disease are, and to deduce the diagnosis from them. This method in diagnosis is preferable to all others, for it leads to greater certainty. At times we may select a few symptoms and let our investigations proceed from them, and we may by this method occasionally get results of considerable certainty; but in general, it may be said that this procedure must be resorted to with caution. The physician should be expert in all methods of investigation. But whatever his special method in given cases may be, his diagnosis should always proceed with a single-eyed regard to the unity of the signs and symptoms, for the diagnosis of the disease is equivalent to the discovery of the relative significance of these. And, again only if he control the technique of the examination, can the diagnostician secure a firm basis upon which to form his diagnosis. An untrained eye cannot discover at once, though it regard the patient ever so sharply, such changes in him as are in contrast with health. In order that the essential points, upon which everything depends, be discriminated from the non-essential, even the eye of the physician must be clinically trained. I am glad to say that the influence of clinical teaching in our colleges has enabled the practitioner to secure for himself a greater precision in diagnosis.

Up to recent times the diseases of the stomach were classified under three heads: those of cancer, ulcer and catarrh. The practitioner was limited in his diagnosis to this conventional classification. Any case that came under his observation was supposed to belong to one of the three in some way or another. For want of any finer differentiation, the diagnosis had to proceed from this traditional and general view. Since Kussmaul, however, things have changed. The introduction of his stomach tube, which he limited at the early stage of it to purposes of treatment alone, has brought about a reformation, not only in therapeutics but also in diagnostics. For as soon as its merits were recognized from its practical side, the step into the scientific and, therefore, larger recognition of it, was inevitable and rapid. By means of the more exact acquaintance with the diseases it addressed itself to, its importance as a help became enlarged upon, by as much as it opened the view into conditions which formerly were closed. At about the time when Ewald improved the somewhat primitive Kussmaul tube, there came into contemporary medicine a scientific revolution by reason of the test methods. These brought out the fact, more than ever before, that functional disturbances of the stomach have a direct relation to pathological conditions. These, it was found, may be of various kinds, and each respective kind could be traced to a pathology of its own. A gastric disorder may be referred to the motility of the stomach, or may be due to changes in the mucous membrane or to anomalies in the secretion of the gastric juice, or to a number of other pathological conditions. And it is possible to recognize these conditions, each of them respectively, and to give to each a therapeusis of its own. They can be differentiated accordingly where as formerly they were not even recognized as having an independent existence.

Taking up one form of stomach disease, namely, chronic catarrh, we may say that it is a very frequent disease and one of the most difficult to diagnose. The literature of the etiology, pathology and the therapeutics of it is extensive, but it is being taken for granted that a sufficient diagnosis of it can be made from such subjective symptoms as loss of appetite, or excessive appetite, dry lips, pasty or salty taste in the mouth, belching of gas, nausea, gaping, sleepiness, sense of oppression, bloated up feeling or feeling of fullness, great pressure in the epigastrium, sense of burning at the pit of the stomach, aversion to work, hypochrondriac symptoms, melancholia, dizziness and headache, constipation, scanty urine, furred tongue, shortness of breath, palpitation of the heart, and a feeling that food stagnates in the stomach. This clinical history would equally apply to gastric neurosis, to erosion, to ulcer, to carcinoma, and many other affections, all of which are difficult to differentiate. For all of these difficulties give us symptoms which are frequently identical, or at least similar. But though we have made a diagnosis of chronic catarrh of the stomach, we have not yet disposed of the diagnosis finally for gastro-enterologists now differentiate between simple mucous, acid and atrophic forms of chronic catarrh. For this they insist upon the repeated microscopical and chemical examinations of the stomach contents after a test breakfast. No physician would make a diagnosis of a kidney lesion without an examination of the urine. Yet a diagnosis of chronic catarrh of the stomach is often made by reputable physicians in no other way than by exclusion. The diagnosis is not so easy as physicians formerly believed, and as some still suppose. I know of nothing so futile as to attempt to make a positive diagnosis of chronic catarrh of the stomach without an examination of the stomach contents. Leube said, "We may content ourselves with the diagnosis of a catarrh of the stomach when we can exclude with some certainty other chronic difficulties which are attended by gastric disturbances." But I may say that such a diagnosis would be of a negative character and therefore not sufficient, surely not in gastric ailments, for we need positive indications for the ascertainment of a precise diagnosis, and by such a one alone we can discriminate between a chronic catarrh of the stomach and affections which resemble it closely. The diagnosis may be assumed upon the ground of clinical symptoms alone, but can be decided *only* after an analysis of the gastric contents. The subjective symptcms, as well as those which are ascertained by the usual methods of examination, do not always present the characteristic phases of the ailment. Analogous phenomena might be observed in all other affections of the stomach. The most experienced physician, if he depend upon clinical symptoms and physical examination alone, might be deceived.

Beaumont taught us from his experience with Alexis St. Martin, as far back as 1830, that the quantity and potency of the gastric juice are affected by the inflammatory condition of the gastric mucosa. In chronic catarrh we have degeneration of the glandular cells on the one hand and connective tissue proliferation on the other. These destroy the function of the cells and lessen the secretion of gastric juice. There are no mucous glands in the stomach, so that normally there should be no mucus present. When masses of glairy mucus are microscopically found in the gastric contents, we have a sure indication of the presence of catarrh. When mucus is entirely absent there is not likely to be any inflammation of the gastric mucosa.

In acute catarrh of the stomach there is an injury to the mucosa and the disturbance of the function of the cells of the glands. We may say that this occurs in a still higher degree in the case of chronic catarrh in which the change in the glandular cells is a permanent one. In chronic catarrh of the stomach we have a deficient fermentation of the gastric juice and the presence of mucus. Because of these, the ingesta are insufficiently digested and undergo fermentation and putrefaction. This can be met if the motility of the stomach is not involved, but experience shows that the musculature suffers in its energy through an inflammatory infiltration. An analysis of the stomach contents will determine this point by an examination with respect to the reduction of hydrochloric acid, pepsin, rennin, the abundant mixtures of mucus and the chemical examination of the ingesta for the putrefactive changes. This, along with subjective symptoms, gives us valuable data. When we have excluded a neurosis, the decrease in the secretion of gastric juice and the presence of glairy mucus are suggestive of catarrh. The secretion of hydrochloric acid is not typical of catarrh, for a diminution of this occurs also in neuroses. A varying degree of it, however, is surely indicative against catarrh, while the presence of increased quantities of mucus, both in the full or empty stomach, indicates chronic catarrh; so also does a noticeable reduction of the secretions of pepsin, and especially of rennin.

The various forms of catarrh can be proved only by an analytical examination of the gastric contents, and their differentiation requires a quantitative as well as qualitative examination; in fact this is the only way we can recognize simple chronic catarrh, chronic mucous catarrh, chronic atrophic catarrh and chronic acid catarrh respectively.

Empiricism in the practice of medicine is rapidly being replaced by rational medicine. Let us use all diagnostic methods we have at our command to ascertain just what we have to combat. In other words, an accurate diagnosis is necessary before our therapeutics can be of any avail. This is more true of diseases of the stomach than of any other. The stomach is a laboratory which prepares the food for absorption or for the action upon it by the juices in the intestine. Just as soon as we know what is taking place in the stomach, it is a very simple matter to assist digestion in a rational way so that our patients will readily respond to the treatment which has a relevancy to their ailment.

*22 Adams Ave. West.*

